# Factors Impacting the Adoption and Potential Reimbursement of a Virtual Reality Tool for Pain Management in Switzerland: Qualitative Case Study

**DOI:** 10.2196/59073

**Published:** 2024-12-04

**Authors:** Josefine Lurtz, Thomas C Sauter, Christine Jacob

**Affiliations:** 1 FHNW - University of Applied Sciences Northwestern Switzerland, Riggenbachstrasse 16, Olten, 4600, Switzerland; 2 University of Oxford, Oxford, United Kingdom; 3 Department of Emergency Medicine, Inselspital, Bern University Hospital, Bern, Switzerland

**Keywords:** eHealth, mobile health, mHealth, digital health, reimbursement, emergency, technology assessment, technology adoption, implementation, VR, virtual reality, pain, experience, attitude, opinion, perception, acceptance, adoption, qualitative, interview, hospital

## Abstract

**Background:**

Pain and its adequate treatment are an issue in hospitals and emergency departments (EDs). A virtual reality (VR) tool to manage pain could act as a valuable complement to common pharmaceutical analgesics. While efficacy could be shown in previous studies, this does not assure clinical adoption in EDs.

**Objective:**

The main aim of this study was to investigate which factors affect the adoption and potential reimbursement of a VR tool for pain management in the ED of a Swiss university hospital.

**Methods:**

Key informant interviews were conducted using in-depth semistructured interviews with 11 participants reflecting the perspectives of all the relevant stakeholder groups, including physicians, nurses, patients, health technology providers, and health insurance and reimbursement experts. The interviews were recorded and transcribed, and the extracted data were systematically analyzed using a thematic analysis and narrative synthesis of emergent themes. A consolidated framework for eHealth adoption was used to enable a systematic investigation of the topic and help determine which adoption factors are considered as facilitators or barriers or as not particularly relevant for the tool subject of this study.

**Results:**

According to the participants, the three key facilitators are (1) organizational environment; (2) tension for change, ease of use, and demonstrability; and (3) employee engagement. Further, the three key barriers to adoption are (1) workload, (2) changes in clinical workflow and habit, and (3) reimbursement.

**Conclusions:**

This study concludes that the adoption of a VR tool for pain management in the ED of the hospital subject of this study, although benefiting from a high tension for change in pain and workload management, is highly dependent on the respective organizational environment, engagement of the clinical staff, and reimbursement considerations. While tailored incentive structures and ambassador roles could benefit initial adoption, a change in the reimbursement landscape and further investigation of the positive effects on workflow effectiveness are required to drive long-term adoption.

## Introduction

### Background

Pain management in emergency departments (EDs) has been shown to be a challenge, with oligoanalgesia, the undertreatment of pain, being a major issue [1], leading to inadequate treatment of pain [2]. According to the European Society for Emergency Medicine, effective pain relief, whether through nondrug methods or medication, involves several key steps: consistently assessing pain levels, using age-appropriate pain management techniques, selecting suitable pain relief medications, understanding potential side effects, and regularly reevaluating both the patient’s pain and their pain management plan to adjust as needed [3]. This implies that addressing acute pain in emergency situations necessitates a personalized approach, taking into account the specific characteristics of each pain management method [4].

Within EDs, pain treatment is impaired by the fact that pain is highly individual, knowledge and education are often scarce, the individual expression of pain is culturally dependent and the workload in EDs is high [1]. While this issue may be addressed through specific education and guidelines on pain treatment [2], tools such as virtual reality (VR) could support clinicians as a complementary pain management option to common pharmaceutical analgesics [5,6]. VR is generally defined as “the use of computer modeling and simulation that enables a person to interact with an artificial 3D visual or other sensory environment. VR applications immerse the user in a computer-generated environment that simulates reality through the use of interactive devices, which send and receive information and are worn as goggles, headsets, gloves, or bodysuits” [7].

VR interventions have emerged as a promising nonpharmacological treatment option for managing pain in patients. Multiple benefits have been demonstrated including decreasing pain intensity perceived, anxiety, unpleasantness, and the time spent thinking about pain during medical procedures [8,9]. Hoffman et al [10] showed that the use of VR tools can result in pain alleviation comparable to a moderate opioid dose, and that it can be an effective complement to common analgesics. As a complementary pain management practice, VR has shown potential for both acute and chronic pain conditions [11]. However, as this study focused on an ED setting, it is centered around acute pain interventions.

The use of VR in pain management is based on the concept of providing immersive, multimodal stimuli to engage the patient, thus distracting patients from their pain [12]. These stimuli may include, but are not limited to, a device, projecting visuals (eg, in the form of a head-mounted display), earphones (potentially noise canceling), and handheld steering devices [12]. The resulting multimodal sensory inputs are an important differentiating factor to passive diversion such as watching television, as the stimuli provide the patient the illusion of being in a different environment [12].

Researchers studying VR as a tool to attenuate pain explain that the underlying methodology of immersive distraction has been identified as a main factor contributing to a reduction in the experienced pain of patients [11]. Hoffman et al [13] argued that VR is redirecting the limited attentional capacity of humans. Thereby, VR treatments would leave less attention available to process and direct input from pain receptors. Strong et al [14] found a similar neuronal explanation, reasoning that neural pathways are not available for transmitting pain signals while occupied with VR input. Gupta et al [11] argue that while there is evidence on VR affecting pain beyond distraction, the respective research is focused on chronic rather than acute pain.

### Objectives

This study investigates the adoption of a VR simulation for pain and anxiety at a Swiss university hospital ED. The VR tool, called Healthymind (HEALTHY MIND), is a patented, CE (conformité européenne) marked, International Organization for Standardization 13485 certified, and registered Class I medical device that is commercially available [15]. The intervention consisted of the application of the Healthymind VR simulation, using a Pico G2 4 K VR headset (Pico Interactive Inc) with a resolution of 1920×2160 pixels and a diagonal field of view of 101 degrees and Bose Quiet Comfort 35 II noise-canceling headphones (Bose Corporation) as an adjunct to usual care in the ED.

The ED team in the hospital subject of this study has conducted an initial research about Virtual Reality for Pain Relief in the Emergency Room to investigate the feasibility of deployment of a VR simulation in the busy setting of the ED for an adult population presenting with traumatic or nontraumatic pain, and the effectiveness of the VR simulation in pain and anxiety control. This work builds on and complements this initial study that was published earlier [5], and TCS shares coauthorship of both papers.

While the tool was introduced with intense training and the highly engaged VR tool advocates pushing adoption, its use has not yet set into clinical routine. Therefore, the key objectives of this work were to assess what factors impact the adoption of the VR tool in question, and whether they act as barriers or facilitators. Furthermore, potential solutions for the identified barriers were also investigated. The authors were guided in their thinking by the consolidated framework for eHealth adoption by Jacob et al [16]. The consolidated framework, informed by the sociotechnical theory [17], categorizes adoption factors in three key clusters: (1) organizational and policy, (2) technical and material, (3) social and personal factors.

## Methods

### Study Design

The qualitative paradigm was chosen due to its emphasis on prioritizing “the voices of participants” and the rich insights it offers [18]. Qualitative methods allow for a deeper understanding of the participants’ individual perceptions in ways that cannot always be attained through quantitative approaches [19].

### Data Collection

Data were gathered through in-depth, semistructured interviews, all held digitally. Additionally, physical artifacts such as screenshots of the VR tool, compatible devices, and examples of user feedback were collected to provide a comprehensive assessment of the tool under study (Healthymind) [20]. The data collection period spanned from March to June 2023, during which a total of 11 interviews were conducted (out of 62 individuals contacted). The interviews were conducted and recorded in German by the first author (JL), with durations ranging from 15 to 60 minutes depending on the participant’s depth of perspective. The interview topic guide is available in Multimedia Appendix 1. Research themes and questions were developed in accordance with Jacob et al’s [16] consolidated framework of the factors impacting eHealth adoption. As per this framework, themes in the interview guide were categorized into three groups: social, organizational, and technical. Data collection continued until a satisfactory level of saturation was achieved, indicating that new data no longer yielded novel insights [19].

### Sampling Techniques and Participant Profiles

Considering the specific expertise required for this research, purposive sampling was used, in which potential participants were selected based on their ability to provide rich and in-depth information about the topic. We used a purposive sampling method, selecting participants based on their capacity to provide detailed and comprehensive information about the app and its use [18,21]. Initially, key informants within the Swiss university hospital ED were approached, and subsequently, snowball sampling was used to identify suitable participants. The primary selection criteria included participants being key stakeholders in the Swiss health care system and in the adoption discussion around VR tools. A “stakeholder” is defined by Effective Health Care Program as a person or group with a vested interest in a particular clinical decision and the evidence that supports that decision [22]; in the context of this study, this includes physicians, nurses, patients, health insurance professionals, policy experts, and employees of the hospital provider of the VR tool and experts of the Swiss Federal Office of Public Health as well as of the cantonal health department. Thereby, this research is incorporating insights from various points of views in the Swiss health care context to portray an inclusive picture on the current situation. Participant recruitment was conducted through tailored LinkedIn and email outreaches, including a detailed study information sheet as shown in Multimedia Appendix 2. Table 1 lists the participant profiles showing their diverse angles that represent the different stakeholders in the Swiss health care ecosystem.

**Table 1.** Participant profiles.

Participant ID	Participant perspective	Organization
P1	Policy expert	Hospital subject of this study
P2	Physician	Hospital subject of this study
P3	Physician	Hospital subject of this study
P4	Nurse	Hospital subject of this study
P5	Technology provider	Provider of the virtual reality tool subject of this study
P6	National policy expert	Member of the Eidgenössischen Kommission für Leistungen und Grundsatzfragen, which translates to Federal Commission for Benefits and Policy Issues
P7	Reimbursement expert	Mandatory insurance provider
P8	National policy expert	From the Federal Office of Public Health
P9	Cantonal policy expert	From the canton where the hospital subject of this study operates
P10	Nurse	Hospital subject of this study
P11	Patient	Hospital subject of this study

### Data Analysis

Thematic analysis was used to identify and extract relevant themes, as well as to interpret their potential meanings and interrelationships [21,23]. For data coding, computer-assisted qualitative data analysis software, specifically Atlas.ti (Lumivero and ATLAS.ti Scientific Software Development GmbH), was used. Excerpts were selected to construct a narrative for each theme, aiding in enhancing comprehension of the analysis. The primary author (JL) conducted the interviews and the analysis and coding. CJ reviewed the coding, and any instances of disagreement were resolved through discussion with the third author (TCS).

**Figure 1.**
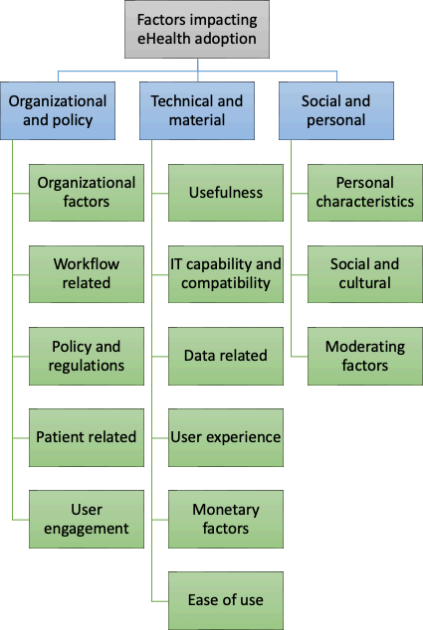
Deductive themes according the consolidated framework for eHealth adoption by Jacob et al [16].

The deductive themes were predefined according to the consolidated framework for eHealth adoption by Jacob et al [16]. Figure 1 shows the deductive themes that served as the starting point for the thematic analysis; the inductive themes emerged from the data as some factors were marked as facilitators, barriers, or not particularly relevant for the case subject of this study.

### Ethical Considerations

All participants signed an informed consent form prior to the qualitative interview. This research was part of an overarching study approved by the local ethics institutional review board Kantonale Ethikkommission Bern (Req-2020‐01,266). All participants were briefed about the research background and signed a consent form agreeing to participate. Participants did not receive any payment for their participation.

## Results

### Overview

Based on the interview analyses, this section synthesizes which factors are currently supporting or inhibiting the adoption of the VR tool for pain management in the ED setting of the Swiss university hospital subject of this study. Multimedia Appendix 3 includes direct quotes from the participants supporting the narrative synthesis of the different themes reported below.

### Organizational and Policy-Related Adoption Factors

#### Organizational Factors

The interview analysis confirms that the overall organizational environment is a critical facilitating factor for adoption. A policy expert within the hospital pointed that the organizational circumstances are indeed beneficial for the adoption of such a technology, as the innovational spirit in a university hospital is high. The physician’s perspective underscores the advantage of university settings in fostering early adoption of new technologies or approaches. Since these environments are often research-focused, financial concerns can take a back seat during pilot phases. This allows for a more exploratory and experimental approach without immediate financial pressures, thus facilitating innovation and the testing of new methods or technologies.

A factor that was unanimously mentioned as a strong facilitator is the inner setting and strong focus around training and education within the hospital, particularly on innovative treatment methods. Physicians and nurses appreciate the promotion of tools such as VR within the hospital and the general focus on new technologies. Specifically, the teaching assignment of the university hospital promotes the adoption of new tools by not merely offering the option to use the device, but by carefully introducing it to the relevant personnel. Therefore, the importance of proper training and the effort required for it were emphasized.

Leadership commitment and management support regarding this and similar projects that go in the direction of digital solutions were explicitly mentioned as a facilitator. Furthermore, participants noted that the VR tool might act as a unique selling point of the organization and its ED in clinician recruitment efforts, which could act as a component reinforcing implementation, especially in the context of a hospital engaging in education.

Lastly, the organizational factor of “tension for change” and the current trend toward such interventions can create traction, as innovations in this direction are anticipated. Additionally, pain management in the ED was confirmed as a pressing concern; having an additional option to alleviate patient pain will facilitate adoption.

#### Workflow-Related Factors

The analysis of workflow-related factors revealed that changes to clinical workflow, workflow fit, resources, and workload are highly interrelated. The required changes in clinical work to implement VR were brought up as a barrier. Workload was mentioned frequently as a main barrier to adoption into the clinical workflow and routine, as time is scarce in the ED.

While VR adoption requires habituation and adaptation, and pharmacological options might initially be faster, a clinician believed that VR may lead to time savings in the long run, benefiting the organization. Although nurses did not explicitly confirm this efficiency gain, the tool could allow them to allocate their time more effectively by distracting patients in pain before further attention is needed. Additionally, the tool’s adoption may support the transformation away from purely pharmacological pain management, which was mentioned as a positive change of clinical care.

Regarding compatibility and adaptability, the provider of the tool emphasized that trials on further adaptation of the software to specific environments or medical uses did not yield better results. That is why they provide environments which are applicable to as many use cases as possible. However, within the possible simulation environments that the provider supplies, customization is possible in a sense of selection of the preferred environment through the patient, which is a facilitating factor.

Although the potential threat of new technologies for clinicians was mentioned during interviews, this does not seem applicable to the VR case at the hospital studied. On the contrary, especially for nurses, this tool is seen as an opportunity to expand their capabilities and skill sets. Additionally, it is beneficial that nurses can decide to use the VR tool without needing to escalate up the hospital hierarchy or require a physician’s prescription.

#### Policy and Regulations

Since the tool is already approved and certified, research participants did not perceive regulatory approval as a barrier, as is often the case in similar studies. During discussions on “policy and regulations,” participants did not raise this point; instead, the focus was on reimbursement aspects, particularly in the context of Swiss health care. From a health insurance perspective, participants emphasized that the Swiss insurance system operates on a fee-for-service payment model, where reimbursement is based on the volume of care services provided. This model incentivizes providers to perform more services, such as filling hospital beds and conducting procedures. Therefore, a solution adds the most value if it increases efficiency, allowing more patients to be seen and generating more billing revenue. This could act as a barrier to adoption for digital health interventions such as the VR tool studied, which focus more on quality and patient experience improvements rather than increasing service volume.

The overarching concern was how the VR application could be funded for patients in pain in the ED. Reimbursement may happen via the mandatory insurance or through a supplementary route. One suggestion for reimbursement through the mandatory health insurance was via the so-called MiGeL (German: *mittel und gegenständeiste* which translates to *means and object list*; Nova Cantica). This exhaustive list for reimbursable tools for the use outside the hospital, was proposed by the federal ministry of health. However, as the VR tool subject of this study is applied inside the hospital, the MiGeL-related reimbursement is not applicable. Instead, reimbursement would happen via TARMED, the current Swiss tariff system for outpatients in hospitals, as the VR tool is to be categorized as a mandatory service provided in the hospital. Such a categorization is not substantiated on an exhaustive list, but by a so-called principle of trust, an assumption that medical insurers reimburse all performed examinations and treatments if they are appropriate according to the WZW criteria (in German: *wirksamkeit, zweckmässigkeit, und wirtschaftlichkeit*; translates to *effectiveness, expediency, and efficiency*). As the categorization as a mandatory service is not currently doubted or questioned by insurers or other parties, reimbursement of mandatory health insurers would be expected. However, the categorization as a mandatory service cannot definitely be confirmed according to the policy experts participating in this study. This categorization would rule out options of reimbursement through complementary insurances, which was the initial reimbursement method suggested by clinicians and payers. Further it rules out self-payment of patients, as mandatory services fall under a so-called tariff protection.

For the categorization as a mandatory service the WZW criteria must be fulfilled. The service provider, in this case the hospital subject of this study and its physicians, must determine whether the WZW criteria are fulfilled by the tool, by demonstrating effectiveness, appropriateness, and economic efficiency. If this can be confirmed, reimbursement within the mandatory health insurance could be given. However, the categorization as a mandatory service through the fulfillment of the criteria above, does neither automatically lead to a cost-covering reimbursement amount, nor does it create tariff positions in the TARMED system for “standard” reimbursement, as TARMED is not kept up to date anymore because it will be replaced in the upcoming years.

Reimbursement could also be pursued through individual case invoices, where a physician would need to justify the necessity and appropriateness of the tool to the insurer. However, this approach is resource-intensive and not advisable in this case. Instead, other short-term solutions should be explored, such as assessing whether the tool’s adoption offers economic advantages, making it attractive even without immediate reimbursement. In the long-term, participating policy experts strongly recommend that the hospital engage in advocacy and help shape the new outpatient tariff system. This would ensure that VR for pain management and other digital tools have an appropriate category within the new system, allowing for seamless reimbursement if categorized as a mandatory service.

#### Patient-Related Factors

Patient access to care was not a concern for the participants, due to the fact that the Swiss EDs are open to all citizens, thanks to compulsory health insurance, anyone living in Switzerland has access to medical care [24]. Similarly, patient safety was not a major concern as the VR tool subject of this study does not prompt any known safety risks according to this study’s participants. However, patient condition and engagement were discussed.

Patient condition was mentioned in the context that the severity of the patients’ pain may impact the tools’ effectiveness, as the necessary immersion may be harder to achieve. While this can have a negative impact on adoption, the general difficulty to appropriately treat the condition “pain” in the ED, including the reluctance for the use of opioids, can be seen as a facilitator, as another treatment option expands the clinicians’ toolkit according to this study’s participants.

The nurses in this study highlighted that patient education requires significant time and effort, which might not always be available. The participating patient confirmed the time taken to inform them and noted their high involvement in deciding whether to use the VR tool. This process substantially increased patient engagement and their willingness to use the tool.

#### User Engagement

Participating physicians emphasized that nurses may perceive the tool as an expansion of their capabilities, which may have a positive effect on their feelings about their job. Confirming this perspective, the interviewed nurses expressed excitement about the application of the new tool. Furthermore, the clinicians explained that fostered engagement may positively affect adoption through the enhancement of employee engagement and other factors such as habit. However, the nurse view also revealed that different nurse team members and physicians individually vary in their perception, which affects teamwork and adoption.

### Technical and Material Adoption Factors

#### Usefulness

The tool’s usefulness was of high relevance for all participants. User perception was generally positive, and the participating clinicians highlighted the great potential of VR for pain management. As for efficiency of care, the picture is more complex. This is as installing and applying the tool takes about 20 minutes, which the care professionals must integrate into their already busy schedule. However, it may be argued that while the patient is occupied with the tool for 20 minutes, the care team may take advantage of this time more efficiently. Therefore, the factor efficiency can be seen as a barrier at a first glance, but it can also be perceived as a facilitator.

Communication was also discussed, with the care team noting that explaining the function and usefulness of the device could take significant time, depending on the patient’s prior knowledge. However, this effort can enhance the patient experience. Both patients and clinicians confirmed that positive stories about the tool increase patients’ willingness to try it. Therefore, clear guidelines on how to effectively present the tool to patients may facilitate its adoption.

#### IT Capability and Capacity

Although IT capability and capacity can affect adoption for certain tools, it was not perceived as of relevance for the VR tool subject of this study as it does not require any additional infrastructure and does not necessitate any integration with other IT systems in the hospital. Even though the hospital subject of this study was going through a major IT transformation at the time of this research, this did not seem to impact use or adoption, which may be due to the tool lacking the need for system integration.

#### Data-Related Factors

Data-related factors were also mentioned as a general challenge for eHealth adoption but were not particularly relevant for the tool subject of the case study, as it does not generate health data. While data quality, exchange, and storage were general considerations shared by policy experts, these were mostly not applicable to the VR tool in question.

#### User Experience

Regarding the design content and quality for patients, the participating nurses highlighted recent software updates of the visual simulation, which made the VR environments more realistic and thereby more immersive and enjoyable. The option to select specific scenarios based on personal preferences was especially appreciated by patients. User experience was generally positive, which suggests good content design and quality. This was further emphasized by the tool’s developer, who shared insights into their design testing and optimization for ideal immersion. One negative aspect which the participating patient brought up, is that certain medical conditions impacting the mobility of the neck, may not be suitable for the application of VR in a lying position, as putting on the headset was experienced as uncomfortable.

#### Ease of Use

High design quality, especially regarding user guidance was indicated by the developer, as they specifically referred to user-centered design and how users are guided through the application step by step on the tablet steering the VR simulation. It was specified that even without training, a person less familiar with technology can handle the device. However, this is partially contradicted by the nurse perspective, which revealed that although the tool was perceived as intuitively usable by some, others had issues using the tool, especially in connection with their personal innovativeness. Specifically, the lack of technical affinity is mentioned to affect ease-of-use on a personal level.

The physician perspective on habit was discussed in relation to using VR tools with patients. The lack of a routine in using VR tools was attributed to workload and time constraints, as conventional medication procedures are habitual and potentially quicker. This issue could be generalized for other innovative treatments. However, the intuitive nature of the VR tool’s handling suggests that it may not pose a significant barrier. From the clinician’s viewpoint, the importance of external encouragement in breaking previous habits and adopting the tool was highlighted, though it was noted that this transition might require ongoing effort.

#### Monetary Factors

While reimbursement potentially finances the use of the device through clinicians and possibly amortizes the equipment costs over time, the initial cost and affordability of the device are not considered, neither are the maintenance and software updates costs. With the limited information at hand, the analysis remains inconclusive whether this factor may act as a barrier, especially as it is highly interrelated with reimbursement considerations.

### Social and Personal Adoption Factors

#### Personal Characteristics

Personal characteristics were indicated to play a relevant role in successful adoption. Participating clinicians explained that patients’ awareness and personal attitudes could be affected through persistent and educative communication. Comfort and acceptance were reported to be positively related to technical skills and experience, and are highly personal. Overall, personal characteristics can therefore be a barrier or a facilitator, depending on the user in question.

#### Social and Cultural Factors

Clinicians’ endorsement is key for the acceptance of the tool and may positively influence individual decisions. The power of endorsement was highlighted by the technology provider and clinicians. It was emphasized that even only one promoting individual physician serving as a VR tool advocate can socially influence others to adopt VR as a tool for patients in pain, especially connected with a push toward a change of habits. Further, the importance of the team was highlighted regarding social influence. When no other team member uses the device, that may therefore inhibit adoption. Conversely, the participating patient voiced that they did not feel impacted by culture or their environment in their decision to use VR for pain management.

#### Moderating Factors

The moderating effects of gender and age were brought up by several participants, implying that younger age was frequently associated with higher comfort and acceptability as well as skills and attitude toward novel technologies. The participating nurses also confirmed that older patients generally require more on-boarding time. Additionally, male gender was associated with a higher openness to try VR for pain management, potentially due to personal experience of gaming and leisure time with VR applications.

## Discussion

### Most Prominent Barriers and Facilitators

#### Overview

The analysis showed that some factors had a more prominent facilitating or inhibiting impact on user adoption than others that had limited relevance for the VR tool subject of this case study. Therefore, the focus of the discussion will be the factors that had a clear impact in this specific case and their respective potential implications for practice.

#### Facilitator 1—Organizational Environment

The organizational environment in which the tool is implemented is considered a critical facilitator to eHealth adoption according to several published studies [25-28], a finding that was confirmed by the outcomes of this research. The participants acknowledged that the environment in the ED of the hospital under study was conducive to adoption. The overall culture of openness to innovation within the hospital was seen as a facilitator, particularly in terms of leadership commitment and the emphasis on training and educating staff in the use of new devices, consistent with findings from similar research [29,30].

#### Facilitator 2—Tension for Change, Ease of Use, and Demonstrability

This study’s participants emphasized that a high tension for change toward more efficient ways of working and pain management is perceived as a facilitator, especially when the adoption of the VR tool allows nurses to focus on other tasks while the patient is immersed in VR and, at the same time, shows clinical effectiveness to reduce pain. Similar studies correspondingly suggested that this demonstrability of the tool’s usefulness, especially in combination with ease of use may indeed positively impact adoption [25,26,31]. Technical affinity has been discussed in relation to the tool’s ease of use; in alignment with similar studies, the easier a technology is perceived in use, the less technical affinity is required [32,33]. In this context, the ease of use of the VR tool under study has been perceived as a facilitator. Furthermore, immediate positive effects on patients after the application regarding anxiety and pain levels could be observed by clinical personnel and could also be proven by the hospital subject of this study [5]. This high level of demonstrability may be used to convince clinicians and patients alike that the VR tool is a valuable, easy-to-use option, especially as the tension for change in pain management within EDs is rising.

#### Facilitator 3—Employee Engagement

The clinicians participating in this study emphasized that employee engagement, particularly because the VR tool is primarily administered by the care team, is a crucial facilitator. Analysis of the interviews suggested that the new tool could have a positive impact on nurses’ attitudes toward their profession by enriching their jobs. This finding aligns with other studies indicating that eHealth tools may empower and expand the roles of health care professionals in certain cases [34,35]. However, further insights revealed that as initial excitement for a technology subsides, this engagement may decrease if not actively managed.

#### Barrier 1—Workload

The first important adoption barrier according to this study’s participants is the experienced workload, especially among nurses that administer the VR tool. As suggested by the literature, workload is a general concern in the health care sector [36,37], and at the same time, health care professionals are expected to increase their work quality and efficiency. This was confirmed by the interviewees to be an inhibiting factor of the implementation of a new tool such as VR for patients in pain. Especially since the tool is currently perceived as an additional task that consumes more time rather than saves time through the set-up of the patient, but especially given the patient education required to ensure willingness to use and overall satisfaction by the patient.

#### Barrier 2—Changes in Clinical Workflow and Habit

A further important barrier that could be observed is the required change in clinical work and therefore in the work habits of nurses and physicians, in alignment with previous studies that emphasized the workflow changes associated with eHealth use as a potential adoption challenge [38,39]. Changing the clinical workflow from the current standard of care’s medication-only approach toward the usage of an additional, complementary tool, requires a substantial adjustment of the standard workflow and work habits of clinicians.

#### Barrier 3—Reimbursement

Finally, the current reimbursement situation in Switzerland is a very important potential barrier for the VR tool’s adoption. The analysis of Swiss reimbursement structures in the outpatient setting of the ED showed that even if the tool fulfills the relevant WZW criteria for reimbursement and is therefore considered a mandatory service under the mandatory health insurance, it could currently not be efficiently reimbursed, given the lack of an appropriate position in TARMED [40]. This is primarily due to the current billing system not being properly maintained as it is to be discontinued [41]. Reimbursing through supplementary insurance is an option, but it would demand significant resources to establish agreements with various commercial insurance companies and would only reach a limited number of potential patients. Moreover, categorizing the tool as a noncompulsory treatment would impede reimbursement through mandatory health insurance in the future, as transitioning from noncompulsory to mandatory service is challenging.

#### Practical Implications

**Figure 2.**
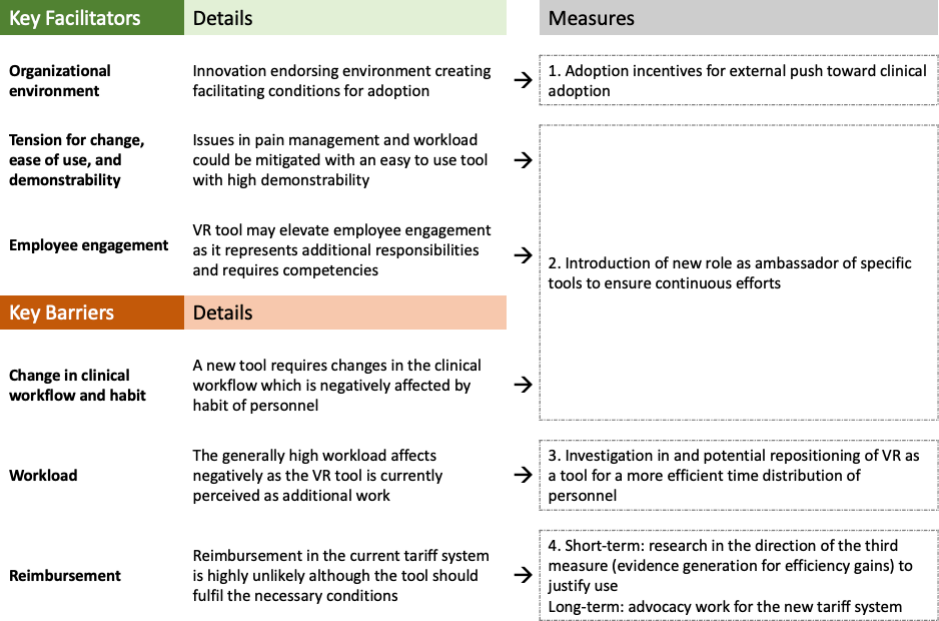
Key facilitators, barriers, and proposed measures. VR: virtual reality.

The analysis revealed a nuanced perspective for some factors, as they may act both as facilitators as well as barriers, depending on the specific circumstances such as personal characteristics of the user (patient and nurse) and future reimbursement landscape. This study’s participants helped us identify three key facilitators and three key barriers. To enhance the impact of the key facilitators and mitigate the effects of the main barriers observed, four measures are proposed as visualized in Figure 2 and explained in the subsequent paragraphs. The numbers on the figure represent the four measures proposed to tackle the corresponding barriers and facilitators.

To foster the beneficial organizational impact, an incentive structure within the ED to drive the adoption of new tools, such as VR for pain management, allows for an external push toward adoption. The introduction of a specific ambassador role is proposed, as a further extrinsic push factor. The ambassador could capitalize on the high tension for change, the ease-of-use of the tool, and the demonstrability as arguments to remind the clinical staff of the valuable option of VR as a complementary pain management option. Moreover, the ambassador could demonstrate and accompany its use to increase employee engagement. Ambassadors could thereby also mitigate the barrier combination of workflow and habit, as their continuous efforts could promote clinical adoption.

To tackle the key barrier of workload, further investigation is proposed in the direction of the potential of the VR tool to support clinical staff in allocating their time as effectively as possible, thereby potentially changing the perception of the tool as additional workload. As patients are immersed in VR, this could potentially lead to less simultaneous attention requirements of the nurse, who is then able to focus on the remaining patients and tasks. Simultaneously, the application could lead to higher patient satisfaction.

Since it is unlikely that reimbursement will improve soon, it is recommended to explore how hospitals can financially benefit from adopting these tools. This could involve assessing their impact on workflow efficiency to see if they reduce workload barriers. This argument suggests that the hospital currently cannot bill the VR service to the patient’s health insurances to achieve reimbursement, but by increasing the effectiveness of the workflows in the ED, the hospital may eventually yield a positive return on investment. However, in the long term, investment in advocacy work by the hospital is suggested to shape the upcoming revision of the Swiss tariff system for outpatient treatment to enable adequate reimbursement of such mHealth tools in the future and have clear positions in the tariff system for VR tools in pain management.

### Limitations and Recommendations for Future Research

This study has some limitations worth noting. We focused on a specific VR tool in one Swiss hospital during a set period of time, so it is hard to generalize our findings to other contexts that might have different characteristics, such as a different regulatory landscape. Although the target was to cover all relevant stakeholder perspectives on the matter, the recruitment of insurance professionals and patients for their perspectives was especially challenging, leading to only one interview for each of those stakeholder types. To minimize the selection bias that can be introduced by purposive sampling, we used snowballing; this involved asking participants to suggest other colleagues who were willing to participate. Lastly, while this research used the comprehensive list of factors in the consolidated framework for eHealth adoption by Jacob et al [16] as a basis for the interview guide and subsequent analysis, it observed connections between factors and disclaimed them when applicable. More research on their interrelatability may be useful when developing measures to address barriers and facilitators. Future studies could explore other tools in different settings to overcome some of these limitations.

### Conclusions

Key facilitators and barriers identified in this study and the respective suggested measures may help improve adoption of the VR tool in the hospital subject of this study. Key facilitators include the supportive atmosphere in settings such as the hospital subject of this case study, which encourages VR adoption and is backed by leadership commitment and training; high demand for change in pain management and VR’s effectiveness and usability, which promote its adoption; and continuous support, which is crucial to sustain user engagement over time.

Key barriers include nurses’ perception that the VR tool is adding to their workload, particularly in patient education and setup; incorporating the VR tool requires the staff to adapt their standard workflows, posing challenges; and the current reimbursement systems lack appropriate codes for VR services, hindering financial incentives.

To address these factors, some measures are recommended: establishing proper incentive structures can encourage VR adoption, ambassador roles can offer ongoing support and advocacy, further research into VR’s impact on workflow efficiency is necessary, and advocacy efforts are needed to influence reimbursement system revisions. By leveraging facilitators and mitigating barriers, hospitals can optimize VR’s benefits in pain management and enhance patient care.

## Supplementary material

10.2196/59073 Multimedia Appendix 1 Interview topic guide.

10.2196/59073 Multimedia Appendix 2 Study information sheet.

10.2196/59073 Multimedia Appendix 3 Direct quotes from the participants supporting the narrative synthesis of the different themes reported in this study (translated from German).

## Abbreviations

CE: conformité européenne
ED: emergency department
MiGeL: mittel und gegenständeiste
VR: virtual reality
WZW: wirksamkeit, zweckmässigkeit, und wirtschaftlichkeit
