# Supplementary material for: Factors Impacting the Adoption and Potential Reimbursement of a Virtual Reality Tool for Pain Management in Switzerland: Qualitative Case Study
Source: JMIR Hum Factors. 2024 Dec 4;11:e59073. doi: 10.2196/59073 (PMC11634046; doi:10.2196/59073)
Supplement: Multimedia Appendix 1 [file humanfactors-v11-e59073-s001.pdf]

VR for pain management

Interview guide version 1 – February 2023

Background Questions

1. Participant introduction

2. What are your personal touchpoints with VR for patient facing pain management?

3. What is your personal perspective on Virtual Reality (VR) for patient facing (pain management)?

Theme 1: Barriers

4. What barriers for adoption do you perceive? *Note all and go through for 5/6*

5. Which are most relevant/impactful and why do you believe so?

6. How could they be mediated/ overcome?

Theme 2: Facilitators

7. What facilitators of adoption do you perceive? *Note all and go through for 5/6*

8. Which are most relevant/ impactful and why do you believe so?

9. How could they be used/ enforced?

IF not covered already **(Theme 3: Reimbursement considerations)**

What is your perspective on the current reimbursement situation of VR tools for pain management? Why? How could reimbursement be accelerated? What are alternative reimbursement solutions?

Special case: patients

Background Questions:

1. Participant introduction (Age, origin, political background)
2. What are your personal touchpoints with VR for patient facing pain management?
3. What was your preconception about the device? (conscious of its existence, skills)
4. Did your perceive that your environment or culture impacted your choice whether to use the device?

Theme 1: Perception

1. How did you perceive your treatment with VR pre, during and post usage?

   PRE

   During

   POST

2. Did you perceive the application of the device as complicated or easy to use?
3. Did the treatment help/ support you?
4. Did you perceive to be included in the decision process on whether to use the device?
5. Did you feel safe? Why?
6. How did you perceive the accompaniment of staff?
7. From your personal experience, is there something that could be improve in the usage of the device?

Theme 2: Reimbursement

8. Would you be willing to pay for the treatment?

9. *IF NO:* What would need to change for you to be willing to pay for the treatment?
