# Supplementary material for: Factors Impacting the Adoption and Potential Reimbursement of a Virtual Reality Tool for Pain Management in Switzerland: Qualitative Case Study
Source: JMIR Hum Factors. 2024 Dec 4;11:e59073. doi: 10.2196/59073 (PMC11634046; doi:10.2196/59073)
Supplement: Multimedia Appendix 2 [file humanfactors-v11-e59073-s002.pdf]

Fachhochschule Nordwestschweiz

Participant Information Sheet

Title of the study:
Factors impacting the adoption and potential reimbursement of a Virtual Reality tool for Pain Management in Switzerland: a qualitative case study.

Background: While international meta-analyses support the efficacy of immersive virtual reality for patient facing pain management solutions, its potential appears to be unused in Switzerland. Therefore, this research aims to find the factors impacting the adoption and potential reimbursement of a virtual reality tool for pain management in Switzerland in the form of a qualitative case study. Beginning with research on the potential of immersive virtual reality in the field, the paper will also give an international outlook on countries where the technology is already used. The thesis will then focus on finding and elaborating on the factors impacting adoption and potential reimbursement through an analysis based on the consolidated framework of impacting clinicians' adoption of mobile health by Jacob et al. This approach is to be complemented by interviews with relevant players in the field.

Objective: The aim of this study is to create a research-based overview on the current barriers and facilitators that can guide the relevant stakeholders to promote the implementation of immersive virtual reality as a standard of care for pain patients in Switzerland.

The high-level research questions that will guide the one-to-one interviews are:

- What are current facilitators for the implementation of IVR for pain patients in the Swiss healthcare environment?
- What are the current barriers for the implementation of IVR for pain patients in the Swiss healthcare environment?
- *If not covered previously;* What is your perspective on the current reimbursement situation of IVR tools for pain management?

Expected outcomes: The resulting barriers and facilitators are aimed to support the key stakeholders in the healthcare ecosystem to further support the implementation of Immersive Virtual reality as a treatment option for pain patients.

Research Team:

The researcher, Josefine Lurtz, is an international Management student with experience in the pharmaceutical industry and strong aspirations within the field of Digital Health.

n|w Fachhochschule Nordwestschweiz

The supervisor, Dr. Christine Jacob, from the University of Applied Sciences Northwestern Switzerland, is a seasoned healthcare expert and researcher, with vast experience in pharma, and healthcare technologies both from practice and research perspectives.

Funding:

This research is financially supported by the Inselspital in Bern.

Participant Information FAQs

1. **Why have I been asked to participate?** Due to your profile being identified as a renowned Digital health expert, Health technology developer, pharma expert, health insurance expert or healthcare professional with expertise in Digital health solutions.

2. You can **refuse to take part** in this study without giving a reason.

3. **What will happen to the results of the study?** The study results may be published in academic journals and presented at conferences.

4. **What will I be asked to do?** You will be invited to a remote interview (preferably via MS Teams). The interview should last about 30-60 minutes and the researcher will ask you questions about the topic explained in the previous section. The researcher is interested in the personal views of the participants as users / experts / developers and not the organizations they are working for. You may be asked for a follow-up interview if appropriate and you are available.

5. **Your participation in the study will be anonymized.** Only the research team will have access to participants' data. No personal or identifiable data be included in the dissemination of the results, which will be anonymized.

6. **Use of quotes** from participants in disseminating the research will be kept anonymous (unless anonymity is explicitly waived by the participant).

7. **Recording equipment** will be used to record the interview.

8. **You can withdraw from the study without** giving a reason. To do so please e-mail me at josefine.lurtz@students.fhnw.ch. The last time it will be possible to withdraw your data is the end of May 2022, given it will not be possible to withdraw once the research analysis and write up has started or findings were potentially published.

9. You do not have to answer any interview questions you do not wish to.

10. **Information that is collected from you** will be securely held. Personal identifiable information (e.g. consent forms) will be kept separately from the data. Participants will

be assigned a study code number and identifying information stored separately from the data.

11. **Courtesy note:** the research budget won’t allow for participant remuneration, however, the research team foresees a relevant value proposition coming from the research results, that may be leveraged by the participants in context of their own work.
