# Supplementary material for: Factors Impacting the Adoption and Potential Reimbursement of a Virtual Reality Tool for Pain Management in Switzerland: Qualitative Case Study
Source: JMIR Hum Factors. 2024 Dec 4;11:e59073. doi: 10.2196/59073 (PMC11634046; doi:10.2196/59073)
Supplement: Multimedia Appendix 3 [file humanfactors-v11-e59073-s003.pdf]

Factor	Sub-factor	Participant quotes
Organizational and policy		
	Organizational factors	“I certainly experienced the funding from the hospital first-hand. I think there is a big focus on working with new technologies and also with virtual reality or extremely medical extended reality. You can see that too. In addition to the attitude at the team lead, who specializes in precisely this, and I think the support there is also very high and the acceptance of such new technologies is also very high.” P3<br><br>“Yes, well, I think we have a clinic here anyway, because we have a lot of good representatives, and they are also interested. They're trained for it and they believe that it works, and I think that's always easy, for example in university hospitals with research. I believe that there is great potential for research, which is why we will certainly need more of it.” P10<br><br>“And on the other hand, it is also the case that in the USA in particular, the opioid crisis, i.e., really the prescription of medication for pain, is more addictive, especially the opioids, so their side effects, criminality, which is partly related. That's why it's nice for us to have another non-medication option at hand.” P2
	Workflow related	“I've already asked but they didn't want to, I don't think they're familiar with it either and unfortunately, I often have to say that I simply don't have the time when we have a really large number of patients and a lot to do. Then I simply don't have the time to take those 10 minutes to install the device. To briefly explain it to the patients.” P4<br><br>“Mhm yes, in general, of course, the whole personnel situation is relatively tense. All the processes, including the administrative processes, are very extensive and very resource-intensive. Accordingly, if a technology could enable a certain level of efficiency in these areas, that would certainly be welcome.” P1
	Policy and regulations	“This also goes back to the idea of quality, i.e., quality is not currently a business case in the Swiss healthcare system. Instead, it's primarily about volume and efficiency. And by their very nature, this is not what these digital solutions are primarily aimed at, but they are actually generally trying to improve the quality of outcomes or the quality of care in some way. But with the current tariffs system, it's difficult to earn money with them. Because that would of course somehow be the second, or an alternative, let's say to an official reimbursement process, if there were simply a system

		in the centers that somehow rewards quality and that remunerates a doctor's practice or the hospital if higher quality is delivered.” P7<br><br>“Applications are not covered in Tarmed. Tarmed is a catalog of services… with eHealth applications the doctor lacks the resources and objects that he uses and can also charge for, be it bandages or something else… these and the remuneration for them are typically shown in the list of resources and equipment.” P8<br><br>“The patient only comes into play or has to pay if I clearly classify the service as not a mandatory service, i.e., if I say that breast augmentation etc. are well-known examples. If I say that this is something that is not a compulsory service according to the KVG, then the patient pays for it themselves, possibly receiving a payment from supplementary insurance, but I charge the patient for it as a non-compulsory service and explain it to them in writing beforehand, i.e., how much does it cost? And then I can charge the patient, but not otherwise.” P1<br><br>“So, the challenge is specifically that there is no specific billing rate for the technical application or app, i.e., it is billed as normal within the scope of a nursing service as well as if the patient, if the nursing staff were to do something else with patients. And that is of course not as financially attractive as always having a specific position. And that's one possibility that I see, whether that would somehow be a possibility in the area of supplementary insurance.” P2
	Patient related	“it's certainly nice to have the option just to relax. Oh, what you see, it's definitely relaxing and so, hmm, can it also distance you from pain? But I'm just in too much pain, so I should have had like a bit less pain to benefit from it. But while I was using it, I realized that it could definitely help.” P11<br><br>“I think especially with patients with very severe pain, it is of course more difficult to surrender and immerse yourself in this new world and I think it depends very much on how well you can surrender and also let go of something.” P3
	User engagement	“And I'm also involved in the VR project with X & X, which gives me the opportunity to expand my repertoire a bit, which I'm very happy about.” P4<br><br>“This tool is really complementary to pharmacology, which is exactly what we need for medication, and I find it very helpful. It certainly helps most patients, that's also written a lot in the study, and our setting is also certainly suitable for that, because the patients come and they already have a stress they often have to wait a long time and that certainly helps not only for the pain.” P10

Technical and material		
	Usefulness	“Well, I'm a big fan of using VR for pain patients. I think it can help a lot and I think there is still a lot of potential that can be exploited.” P3<br><br>“…it would of course be great if you could shorten it *(the preparation time)* to 5 minutes, but I don't think that's realistic in that you have to pack a lot into it and this preparation time for patients is just there for the information, there is also the whole preparation of the technology.” P4<br><br>“I think if we already share that it works, that the other patients have experienced it as positive and how we explain it or that we can simply take away the fear.” P10
	IT capability and compatibility	“So, at this point in time, the infrastructure, IT, infrastructure, internal processes, which of course could also be seen as a barrier if internal processes block it.” P1<br><br>“I think the second major hurdle lies in the nature of the healthcare system. It's simply incredibly fragmented, and that makes it very, very difficult to scale solutions… In other words, as soon as I actually have to integrate somewhere with other solutions in the practice information systems, in the hospital information systems, which is usually the prerequisite for any application in the practice.” P7
	Data relaed	“On the other hand, things like AI, right? Basically, anything to do with outsourcing data. Almost a hurdle in the genetics sector too. How do we now handle this genetic data so that we also have the trust of doctors? … I didn't mention data protection as a hurdle, but that can actually be overcome.” P8
	User experience	“We've just got the latest software update on our device, so it's no longer these cartoon worlds, I'd say, but real cities where you can look around. That's really cool.” P4<br><br>“Exactly like a hypnosis therapist would do. I mean same disconnection the association. And reassociation principle that is used like a medical diagnosis, plus the fact you have a headphone with the views, the noise reducing active noise reducing headsets. So, for example, both headsets which really reduce noise and put the patient inside the bubble. So, he doesn't hear the surrounding anxiety property, provoking a noise.” P5<br><br>“I prefer if you can put it on and take it off yourself, but that might only be useful if you use it several times for pain therapy. It was great that I could also select programs. What I would like to see.” P11

	Monetary factors	"From a cost perspective this tool is certainly not as expensive as some others, but of course you can't ignore all the software, all the updates that have to be carried out, and so on." P1
	Ease of use	"…we try to make it very easy to use. So even without training, even person the less familiar with technology can handle the device. It's so, there is a tablet connected now where wireless to the asset and on this tablet all steps are guided and we tell the caregiver what to do. Well, so we try to make it as a user friendly as possible. Um, so typically in anybody can use it there. They don't have to have any special qualifications, so that's definitely helping." P5<br><br>"But I know that I've introduced a lot of people to this technology and I've noticed that many of them find it difficult. But it's a tablet. There are 2 cables, there are headphones, and there are these glasses and all of that has to be combined somehow and that was already too much and I think that's perhaps also the inner attitude. If you're interested in it, then you're also interested in how the technology works and if you don't really want that, then you don't want to deal with the technology at all and then you find it too much." P4
Social and personal		
	Personal characteristics	"I think it also depends a lot on the person. There are doctors who are also very tech-savvy, they think about it themselves. Sometimes they even suggest it. And then there are others who don't think about it at all. Maybe they haven't been trained. That can also be the case. And then you can bring it up with them. Yes, and it always depends a bit on the doctor and it's often my decision whether or not to offer it to my patients, as long as it doesn't hinder anything, i.e., doesn't hinder any further examinations or therapy, I can do it myself, so I don't need the doctor to give their OK in that sense. I can decide that." P4<br><br>"At first the patient was a little skeptical, then we explained it to her and she used it and to be honest it didn't help that much with the pain, but it really helped her to relax, to take away a little of the anxiety and I think that's really great. On the VR glasses, because that doesn't just work on the pain, but really also on the other stress factors or anxiety" P10
	Social and cultural	"So, the of all facilitator, the one which is on the very much more powerful is to have one person that's really be will be the referent of the VR and will believe in it so much at that it will push to its use. It will really, make the use habits in the process of care in the department, it doesn't need to be head of department, doesn't need to be a doctor or anything. Can be anyone, so a nurse or and a doctor as well. It can be anybody, but someone." P5

		“Yes, it is certainly a barrier if VR technology is not promoted as it is now at our hospital, so I don't know enough about other clinics, but I could imagine if they don't have a driving force that really actively introduces and promotes it, it won't get off the ground.” P3
	Moderating factors	“What I've noticed is that young patients, for example, rather young male patients, do I have to say, are more likely to get involved? Because maybe it's a different thing now, maybe the patients that are familiar with gaming are more familiar with the technology and they find it interesting” P4<br><br>“Maybe the acceptation is even higher for younger people. So, for children, they are even more into it, and the acceptation rate is getting a bit lower for elderly people.” P5
